# Spatiotemporal patterns of male and female white‐tailed deer on a hunted landscape

**DOI:** 10.1002/ece3.9277

**Published:** 2022-09-11

**Authors:** Dylan G. Stewart, William D. Gulsby, Stephen S. Ditchkoff, Bret A. Collier

**Affiliations:** ^1^ College of Forestry, Wildlife and Environment Auburn University Auburn Alabama USA; ^2^ School of Renewable Natural Resources Louisiana State University Baton Rouge Louisiana USA

**Keywords:** fear, hunting, predation risk, resource, selection, sexual segregation

## Abstract

Resource selection in sexually dimorphic ungulates is at least partially explained by sex‐specific resource requirements and risk aversion strategies. Females generally spend more time in areas with less risk and abundant, high‐quality forage due to their smaller body size. However, demographically variable responses to risk are context dependent, and few have concurrently quantified male and female behavior within areas with the same resource base. We captured 111 (54 males, 57 females) adult white‐tailed deer (*Odocoileus virginianus*) from 2009 to 2018 on a site in South Carolina, USA, where hunters were the primary source of adult mortality. We fit each deer with a GPS collar programmed to collect locations at 30‐min intervals. Upon collar recovery, we analyzed the data to estimate sex‐ and time‐specific selection for, and distance to, various cover types. While both sexes generally avoided risky areas (i.e., sites hunted more frequently) during the day, females (*p* = .41) were more likely than males (*p* = .16) to use risky areas containing abundant food resources during the day, where *p* = probability of selection. Our findings indicate that female white‐tailed deer may be forced to utilize high risk areas during high risk periods due to their smaller body size and increased nutritional demands, whereas larger males are better able to forgo foraging opportunities during risky periods to mitigate risk; however, our study design left room for the possibility that our observations were driven by innate sex‐specific patterns in white‐tailed deer. Nonetheless, our study contributes information to the literature by describing sex‐specific resource selection by diel period on a site where sexes shared the same resources and were presented with the same landscape of risk.

## Graphical abstract

Prey species must balance their need for food acquisition with predation risk. In a hunted population of white‐tailed deer, males avoided risky areas containing abundant food during times when hunters were more likely to be present. However, females were more likely to visit these risky areas when hunters were present, likely because physiological and energetic demands of raising young require them to access higher quality forage.

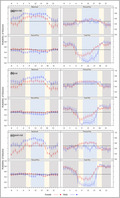

## 1 INTRODUCTION

Resource selection by animal species is influenced by several factors, including balancing forage acquisition with predation risk (Bowyer, 2004; Ruckstuhl & Neuhaus, 2002). When an area is both risky and forage rich, animals often shift use temporally as a risk aversion measure (Creel et al., 2008), but risk‐driven behavioral decisions may differ between sexes for sexually dimorphic ungulate species. Specifically, females generally require greater quality forage than males due to their smaller body size and shorter food passage times (Berini & Badgley, 2017), and higher quality female diets have been documented for an array of ungulate species (Beier, 1987; Berini & Badgley, 2017; Long et al., 2009; Luna et al., 2013; Oehlers et al., 2011). Sex‐specific body size differences and corresponding predation risk (Bleich et al., 1997; Main et al., 1996; Oehlers et al., 2011), or the presence of young (typically with adult females; Higdon et al., 2019), may also affect resource selection.

In addition, sex‐specific responses to risk are context dependent, varying according to time of year (and corresponding vulnerability), predator community, and landscape composition (Bleich et al., 1997; Crawford et al., 2019; Festa‐Bianchet, 2012; Pérez‐Barbería et al., 2005). For example, male deer may show stronger predator avoidance than females during times of the year when males are more vulnerable to predators (Cherry et al., 2015). In areas where human hunters are the primary source of adult mortality, both sexes of white‐tailed deer reduce risk of hunting mortality by limiting activity and using areas that provide concealment during the day (Bakner et al., 2020; Henderson et al., 2020; Karns et al., 2012; Little et al., 2016; Sullivan et al., 2018). Similar patterns have been documented for both roe deer (*Capreolus capreolus*; Cimino & Lovari, 2003, Bonnot et al., 2013) and fallow deer (*Dama dama*; Borkowski & Pudelko, 2007) in hunted systems. However, in systems with primarily nocturnal predators (i.e., Florida panthers [*Puma concolor coryi*]) white‐tailed deer have been shown to do the opposite, by increasing daytime activity (Crawford et al., 2019).

Despite the abundance of information on factors affecting resource selection in sexually dimorphic ungulates, most studies have focused on one sex (Henderson et al., 2020; Little et al., 2014; Little et al., 2016; Marantz et al., 2016; Shuman et al., 2018; Sullivan et al., 2018), one aspect of deer behavior (e.g., movement characteristics or resource selection; Cherry et al., 2015, Biggerstaff et al., 2017), or in locations where risk was primarily driven by nonhuman predators (Crawford et al., 2019; Higdon et al., 2019). Therefore, our objective was to concurrently describe spatiotemporal patterns of resource selection by both sexes of a sexually dimorphic ungulate species on a site where human hunters were the primary source of adult mortality. Based on the previous literature, we hypothesized that both sexes would select for areas with greater concealment cover and avoid hunted areas during the day, and select for areas with more abundant forage at night. However, we expected females would be more likely to use risky, forage‐rich areas during the day than males due to their greater nutritional requirements.

## 2 METHODS

We conducted our research at Brosnan Forest, a 5828‐ha private property owned and managed by Norfolk Southern Railroad within the Coastal Plain ecoregion in Dorchester County, South Carolina, USA. Research activities were limited to the 2593‐ha portion of the property located north of Highway 78. Brosnan Forest was 93% forested, consisting of four dominant cover types: natural pine, planted pine, hardwood drains, and food plots. Four additional cover types (bottomland hardwood, clearcut, lake, wet area) represented the remaining ~5% of the study area. Natural pine stands were dominated by ~120‐year‐old loblolly (*Pinus taeda*) and longleaf (*P. palustris*) pine trees, managed with commercial thinning to maintain an open canopy. Low‐intensity prescribed fire was applied every 2–3 years to natural pine stands to maintain a relatively open understory dominated by herbaceous plants (Collier et al., 2007; Lauerman, 2007). Natural pine stands covered 1564 ha (~60% of the study site). Planted pine stands covered 500 ha (19%) of the study area and consisted of ~20‐year‐old loblolly and longleaf pine trees with closed canopies. Hardwood drains covered 258 ha (10%) of the study area, and consisted of closed canopy forests dominated by a variety of oaks (*Quercus* spp.), sweetgum (*Liquidambar styraciflua*), red maple (*Acer rubrum*), and pond cypress (*Taxodium ascendens*). Food plots ranged in size from 0.03 to 8.5 ha (150 ha total; 6% of the study site), and were planted annually with a cool season mix of various clovers (*Trifolium* spp.), oats (*Avena sativa*), wheat (*Triticum aestivum*), chicory (*Cichorium intybus*), and winter peas (*Pisum sativum*), while others were planted in warm‐season crops including soybeans (*Glycine max*), sorghum (*Sorghum bicolor*), buckwheat (*Fagopyrum esculentum*), benne (*Sesamum indicum*), and sunflowers (*Helianthus* spp.; Sullivan et al., 2018). Additionally, there were ~ 60 game feeders at a density of ~1 feeder/50‐ha, primarily located within food plots, throughout the study site that dispensed shelled corn during our study (Goethlich, 2020; Sullivan et al., 2017). There were 153 km of roads used for transporting hunters throughout the site.

The male to female sex ratio for the property was previously estimated at 1:1.4 (Sullivan et al., 2017), and the male to female sex ratio of hunter harvest during our study was 1:2, with females making up an average of ~65% of the 425 (range = 234–506) deer harvested annually (McCoy et al., 2013). Additionally, hunters were encouraged to practice quality deer management by harvesting deer ≥3.5 years old, with ~60% of males being ≥3.5 years old at harvest (McCoy et al., 2013). The deer hunting season on Brosnan Forest was from 15 September to 1 January, with hunters being transported either mornings (~6:00–9:00) or evenings (~16:00–19:30) to permanent stand locations overlooking food plots or private, internal roads (Sullivan et al., 2018; C Brownlee, personal communication). Average hunting effort was 5 h 100 ha^−1^ week^−1^, which could be considered low‐risk (Little et al., 2014), and hunters were frequently rotated throughout different portions of the property, occupying only 10% of stands daily, to minimize disturbance at each location (Sullivan et al., 2018).

We determined the area of vulnerability of deer to hunters in each cover type by sitting in each stand prior to the hunting season and using a laser rangefinder to determine the area within a 100‐m radius in which deer would be visible to a hunter (Sullivan et al., 2018). We then created area‐of‐vulnerability polygons for each stand location, overlaid these on the cover type layer, and used the tabulate intersection tool in ArcMap 10.2 (ESRI) to determine the proportion of each cover type in which deer were susceptible to hunters. We then compared the proportion of each cover type in which deer were viewable by hunters to the composition of areas surrounding stands to determine relative risk to deer in each cover type. The area surrounding fixed hunting stands was roughly comprised of the same proportion of each cover type as the study area, except for food plots, which covered 6% of the study area, compared to 15% of the area around stands. However, the area in which a deer could be viewed by a hunter from a stand was comprised of 23% food plot (approximately four times greater than availability across the study area), 4.5% hardwood drain (less than half of availability across the study area), and 13% planted pine (compared to 19% across the study area).

Additional disturbance (i.e., forest management, Northern Bobwhite [*Colinus virginianus*] hunts, feeder maintenance, and plantings) could also be considered low and dispersed throughout the property, primarily occurring between the hours of 7:30 AM–4:30 PM daily (C. Brownlee, personal communication). Predator trapping began in 2003 following the range‐wide expansion of coyotes (*Canis latrans*) into South Carolina in the 1990s (Hody & Kays, 2018; McCoy et al., 2013). The trapping season ran from January through April, with ~107 individuals (i.e., bobcats [52.1%; *Lynx rufus*], coyotes [36.5%], feral dogs [11.4%; *Canis lupus familiaris*]) being removed annually (McCoy et al., 2013). The fawn predation rate for the property was estimated at 13.8% (29 of 210 fawns; McCoy et al., 2013), which is uncharacteristically low for the Southeast (Kilgo et al., 2019).

During May–August of 2009–2011, 2013–2015, and 2017–2018, we chemically immobilized adult (1.5–4.5+ years old) white‐tailed deer via a 2‐cc transmitter dart (Pneu‐dart Inc.) containing a mixture of Xylazine (Lloyd Laboratories, Shenandoah, Iowa; 100 mg/ml given at a rate of 2.2 mg/kg) and Telazol (Fort Dodge Animal Health, Fort Dodge, Iowa; 100 mg/ml given at a rate of 4.5 mg/kg; Sullivan et al., 2018). We fitted deer with an ATS G2110D GPS Collar (Advanced Telemetry Systems) positioned upright and tightened to allow a ~3 cm gap between the collar and the neck. Post processing, we administered a 3‐ml intramuscular injection of Tolazoline (Lloyd Laboratories, Shenandoah, Iowa; 100 mg/ml given at a rate of 6.6 mg/kg) to act as a reversal to the sedative. We closely monitored deer until they regained functionality and moved away freely. All procedures were approved by the Auburn University Animal Care and Use Committee (PRN no. 2008‐1489, 2013‐2205, 2017‐2996).

We programmed GPS collars to take fixes at 30‐min intervals (48 fixes/day) from 23 August to 23 November. Each fix recorded the individual's location in UTM coordinates, date, time, satellites, fix status, position dilution of precision (PDOP), horizontal dilution of precision (HDOP), and ambient temperature. Upon retrieving data from recovered collars, we removed likely erroneous 3‐dimensional fixes with PDOP >10 or HDOP >6, and 2‐dimensional fixes with HDOP >3 from the dataset (D'Eon & Delparte, 2005; Lewis et al., 2007; Sullivan et al., 2017; Sullivan et al., 2018).

We classified GPS fixes as occurring within one of three seasons: pre‐rut (23 August–18 September), rut (19 September–28 October), and post‐rut (29 October–23 November), with the rut period encompassing 80% of conceptions previously determined on the study site (Byrne et al., 2014; McCoy et al., 2013; Sullivan et al., 2016; Sullivan et al., 2017). We also classified each position according to the time of day it was collected using sunrise and sunset times for the property (National Oceanic and Atmospheric Administration, 2022). To account for the crepuscular nature of white‐tailed deer, we considered any position taken from 30 min before sunrise to 2 h post‐sunrise as dawn, any position taken 2 h before sunset to 30 min post‐sunset as dusk, positions recorded between dawn and dusk as day, and positions recorded between dusk and dawn as night.

We created a GIS layer for each dominant cover type (i.e., natural pine, planted pine, hardwood drain, and food plot) using ArcMap 10.2 (ESRI Inc.). We then overlaid all GPS fixes on the cover type layer and censored the data using a two‐step approach. First, we censored fixes for which cover‐type data were unavailable (i.e., outside the study site; Kroeger et al., 2020). Second, only individuals with a fix success rate ≥80% per season (pre‐rut [≥1037 fixes], rut [≥1536 fixes], post‐rut [≥998 fixes]) were included in the analysis to avoid bias associated with data loss (D'Eon et al., 2002; Frair et al., 2010; Godvik et al., 2009).

We analyzed selection for each dominant cover type using a resource selection function (RSF), in which probability of use was defined as the proportional use of that cover‐type relative to its availability within the home range, resulting in a third‐order selection (Aebischer et al., 1993; Boyce et al., 2002; Johnson, 1980; McKee et al., 2015; Morano et al., 2019). Specifically, we used functions within the adehabitatHR package (Calenge, 2006) in R statistical software (version 4.0.2, R Core Team, 2020) to create 95% kernel home ranges, and functions within the raster package (Hijmans et al., 2020) to extract our covariate data (Karns et al., 2012; McKee et al., 2015). Within each deer home range, we generated random locations using the sp package (Pebesma & Bivand, 2005) at a ratio of 1:1 to the number of used locations for that individual (D'Eon, 2003; McKee et al., 2015). Random and used point locations were generated for dawn, day, dusk, and night periods per breeding season per individual. We used generalized linear mixed‐effects models (GLMMs) within the package lme4 (Bates et al., 2014) to estimate probability of resource selection relative to its availability within the home range (Benson et al., 2016; Johnson, 1980), and used Akaike's Information Criterion, adjusted for small sample size (AICc), to evaluate the relative support for each of our eleven candidate models using the AICcmodavg package (Mazerolle & Mazerolle, 2017). We avoided pseudoreplication and inflated sample‐size issues by adding individual and year as random effects (Aebischer et al., 1993; Otis & White, 1999; White & Garrott, 1990). Model‐predicted selection values (probability values) were calculated using the ggeffects package (Lüdecke, 2018).

We analyzed sex‐specific movement rates by season and time of day. Specifically, we used the package adehabitatLT (Calenge, 2006) to create movement trajectories and the package move (Kranstauber et al., 2020) to structure movement data for analysis. We defined rate of movement (m/0.5 h) as the distance between two consecutive GPS fixes (Sullivan et al., 2018; Webb et al., 2010). Additionally, we used the sp and move packages to determine turn angles. We omitted data with time intervals between two consecutive points greater or less than 0.5 h (±0.08 h). We used mixed‐effect analyses of variance models (ANOVA) within the nlme package (Pinheiro et al., 2017) to estimate rate of movement (m/0.5 h) of all individuals and used AICc to evaluate relative support for each of our eleven candidate models. Model predicted rate of movement values were calculated using the ggeffects package.

We used functions within the sf package (Pebesma & Bivand, 2005) in R statistical software to quantify the distance (m) between each GPS fix and the closest food plot and road, which we anticipated would represent areas of high risk (Bonnot et al., 2013; Kilgo et al., 1998). We used mixed‐effect analyses of variance models (ANOVA) to determine the minimum distance (m) between each GPS fix and areas of risk and used AICc to evaluate the relative support for each of our seven candidate models. Model‐predicted distance to areas of risk values were calculated using the ggeffects package.

## 3 RESULTS

We collected 291,033 GPS locations from 70 GPS tagged individuals (42 females, 28 males) with ≥80% fix success rates throughout each season of our study. The average age at time of capture was ~3.2 years for females, and ~2.5 years for males.

**FIGURE 1:**
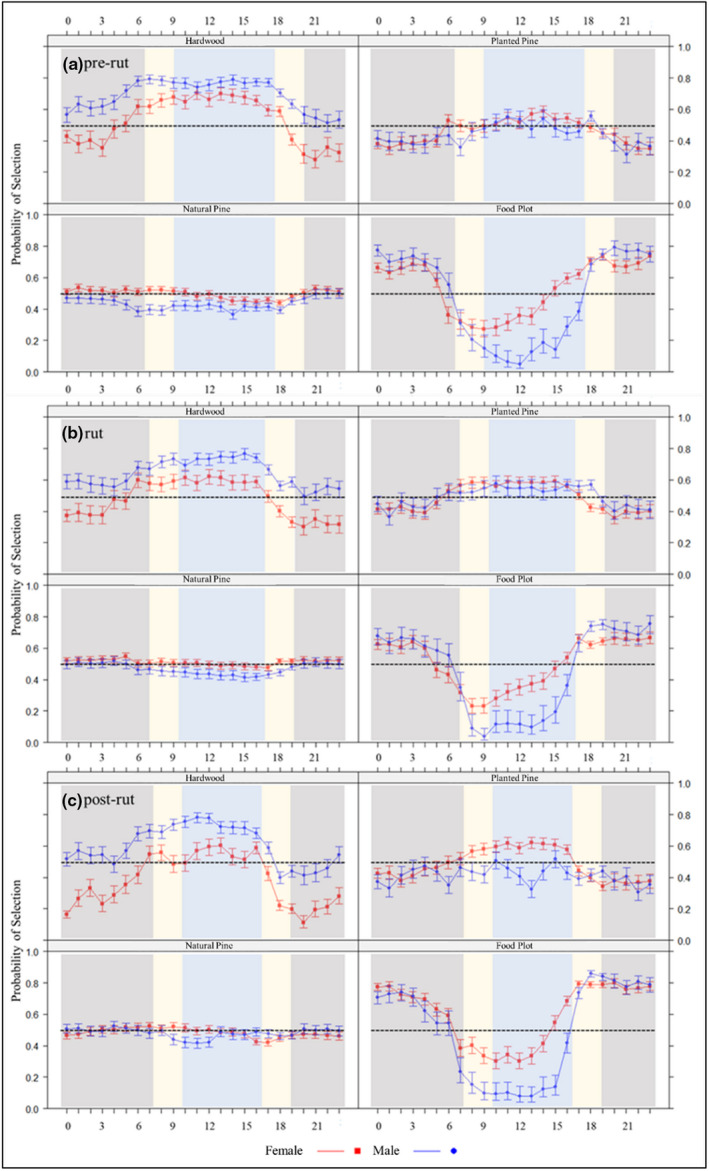
Effect of sex, hour of day, and season on white‐tailed deer (Odocoileus virginianus) probability of selection for various cover types from 2009 to 2018 in South Carolina, USA. Dark gray, beige, and light blue bands are considered night, dawn or dusk, and day, respectively. Error bars represent 95% confidence intervals.

Our top resource selection model included a four‐way interaction among cover type, sex, time of day, and season (Table 1). During the day, where *p* is the probability of use, males selected for hardwood drains over any other cover type during all three periods (*p* = .71), and planted pines were second most selected (*p* = .55; Figure 1; Table 2). Females also selected for hardwood drains during the day (*p* = .61) but selected more strongly for planted pines during the post‐rut period (*p* = .62). Female selection for food plots during dawn (*p* = .29), day (*p* = .41), and dusk (*p* = .71) was greater compared to males during dawn (*p* = .22), day (*p* = .16), and dusk (*p* = .69). Similarly, both males and females had greater selection for food plots at dusk (male: *p* = .69; female: *p* = .71) compared to dawn (male: *p* = .22; female: *p* = .29). Overall, differences in selection across cover types were less segregated for females than for males during the day. Both sexes preferred food plots (male: *p* = .72; female: *p* = .65; Table 2) over all other cover types at night.

**TABLE 1:** Number of parameters (K), Akaike's Information Criterion (AIC_c_), difference from lowest AIC_c_ (ΔAIC_c_), and model weights (w) for candidate models used to predict the effects of sex, time of day, and period of the breeding season on probability of selection for various cover types by white‐tailed deer (*Odocoileus virginianus*) within the home range from 2009–2018 in South Carolina, USA.

Candidate model	K	AIC_c_	ΔAIC_c_	W
Cover type * sex * time * breeding season	98	217778.92	0.00	1.00
Cover type * sex * time	34	218132.07	353.15	0.00
Cover type * time * breeding season	50	218567.41	788.49	0.00
Cover type * sex * breeding season	26	221356.67	3577.75	0.00
Cover type + sex	7	222155.91	4376.99	0.00
Cover type + time	9	222157.45	4378.54	0.00
Cover type + sex + time	10	222159.38	4380.46	0.00
Cover type + sex + breeding period	9	222159.49	4380.57	0.00
Cover type + time + breeding season	11	222161.03	4382.12	0.00
Cover type + sex + time + breeding season	12	222162.96	4384.04	0.00
Null	3	223680.64	5901.73	0.00

**TABLE 2:** Mean estimates (β), standard errors (SE), lower confidence limits (LCL), and upper confidence limits (UCL) predicting the effects of sex, period of the breeding season, time of day, and cover type on probability of selection (*p*) of white‐tailed deer (*Odocoileus virginianus*) within the home range from 2009 to 2018 in South Carolina, USA.

Sex	Season	Time	Cover type	β	SE	LCL	UCL
Male	Pre‐rut	Dawn	Food plot	0.30	0.13	0.25	0.36
			Hardwood	0.75	0.06	0.73	0.77
			Natural pine	0.41	0.05	0.39	0.43
			Planted pine	0.53	0.07	0.49	0.57
		Day	Food plot	0.15	0.18	0.11	0.21
			Hardwood	0.73	0.07	0.70	0.76
			Natural pine	0.42	0.05	0.39	0.44
			Planted pine	0.59	0.07	0.55	0.62
		Dusk	Food plot	0.64	0.10	0.59	0.68
			Hardwood	0.68	0.07	0.65	0.70
			Natural pine	0.42	0.05	0.40	0.45
			Planted pine	0.54	0.07	0.50	0.57
		Night	Food plot	0.75	0.09	0.71	0.78
			Hardwood	0.58	0.07	0.54	0.61
			Natural pine	0.48	0.04	0.46	0.50
			Planted pine	0.42	0.08	0.38	0.46
	Rut	Dawn	Food plot	0.21	0.17	0.16	0.27
			Hardwood	0.71	0.07	0.68	0.73
			Natural pine	0.45	0.04	0.42	0.47
			Planted pine	0.55	0.07	0.52	0.59
		Day	Food plot	0.16	0.19	0.12	0.22
			Hardwood	0.72	0.07	0.70	0.75
			Natural pine	0.43	0.05	0.41	0.46
			Planted pine	0.55	0.07	0.51	0.58
		Dusk	Food plot	0.66	0.10	0.61	0.70
			Hardwood	0.66	0.07	0.63	0.69
			Natural pine	0.44	0.05	0.42	0.46
			Planted pine	0.54	0.07	0.50	0.57
		Night	Food plot	0.68	0.10	0.64	0.72
			Hardwood	0.56	0.07	0.52	0.59
			Natural pine	0.51	0.04	0.49	0.53
			Planted pine	0.43	0.08	0.39	0.46
	Post‐rut	Dawn	Food plot	0.14	0.19	0.10	0.19
			Hardwood	0.69	0.07	0.66	0.72
			Natural pine	0.48	0.04	0.46	0.50
			Planted pine	0.50	0.07	0.46	0.53
		Day	Food plot	0.16	0.19	0.12	0.22
			Hardwood	0.69	0.07	0.66	0.72
			Natural pine	0.47	0.04	0.45	0.49
			Planted pine	0.52	0.07	0.48	0.55
		Dusk	Food plot	0.77	0.09	0.73	0.80
			Hardwood	0.55	0.08	0.52	0.59
			Natural pine	0.47	0.04	0.45	0.49
			Planted pine	0.47	0.08	0.43	0.50
		Night	Food plot	0.74	0.10	0.70	0.78
			Hardwood	0.52	0.08	0.49	0.56
			Natural pine	0.51	0.04	0.48	0.53
			Planted pine	0.41	0.08	0.37	0.45
Female	Pre‐rut	Dawn	Food plot	0.30	0.07	0.27	0.33
			Hardwood	0.64	0.06	0.61	0.66
			Natural pine	0.52	0.03	0.50	0.53
			Planted pine	0.52	0.05	0.49	0.54
		Day	Food plot	0.43	0.07	0.40	0.46
			Hardwood	0.66	0.07	0.63	0.69
			Natural pine	0.48	0.03	0.46	0.50
			Planted pine	0.54	0.05	0.51	0.57
		Dusk	Food plot	0.70	0.06	0.68	0.72
			Hardwood	0.51	0.07	0.48	0.55
			Natural pine	0.46	0.04	0.44	0.48
			Planted pine	0.49	0.06	0.46	0.51
		Night	Food plot	0.63	0.06	0.61	0.66
			Hardwood	0.41	0.08	0.37	0.45
			Natural pine	0.52	0.03	0.51	0.54
			Planted pine	0.40	0.06	0.37	0.43
	Rut	Dawn	Food plot	0.26	0.09	0.23	0.29
			Hardwood	0.60	0.07	0.57	0.63
			Natural pine	0.51	0.03	0.49	0.53
			Planted pine	0.57	0.05	0.55	0.60
		Day	Food plot	0.40	0.08	0.36	0.43
			Hardwood	0.60	0.07	0.57	0.63
			Natural pine	0.49	0.03	0.48	0.51
			Planted pine	0.57	0.05	0.54	0.60
		Dusk	Food plot	0.66	0.06	0.64	0.69
			Hardwood	0.44	0.08	0.41	0.48
			Natural pine	0.50	0.03	0.48	0.51
			Planted pine	0.45	0.06	0.42	0.48
		Night	Food plot	0.60	0.06	0.57	0.63
			Hardwood	0.42	0.08	0.38	0.46
			Natural pine	0.52	0.03	0.51	0.54
			Planted pine	0.42	0.06	0.39	0.45
	Post‐rut	Dawn	Food plot	0.32	0.08	0.28	0.35
			Hardwood	0.57	0.07	0.53	0.60
			Natural pine	0.51	0.03	0.50	0.53
			Planted pine	0.56	0.05	0.54	0.59
		Day	Food plot	0.40	0.08	0.36	0.44
			Hardwood	0.56	0.07	0.53	0.60
			Natural pine	0.48	0.03	0.46	0.50
			Planted pine	0.62	0.05	0.60	0.64
		Dusk	Food plot	0.77	0.06	0.75	0.79
			Hardwood	0.43	0.08	0.39	0.47
			Natural pine	0.42	0.04	0.41	0.44
			Planted pine	0.46	0.06	0.43	0.48
		Night	Food plot	0.73	0.06	0.71	0.75
			Hardwood	0.35	0.09	0.31	0.39
			Natural pine	0.48	0.03	0.47	0.50
			Planted pine	0.40	0.06	0.37	0.43

**FIGURE 2:**
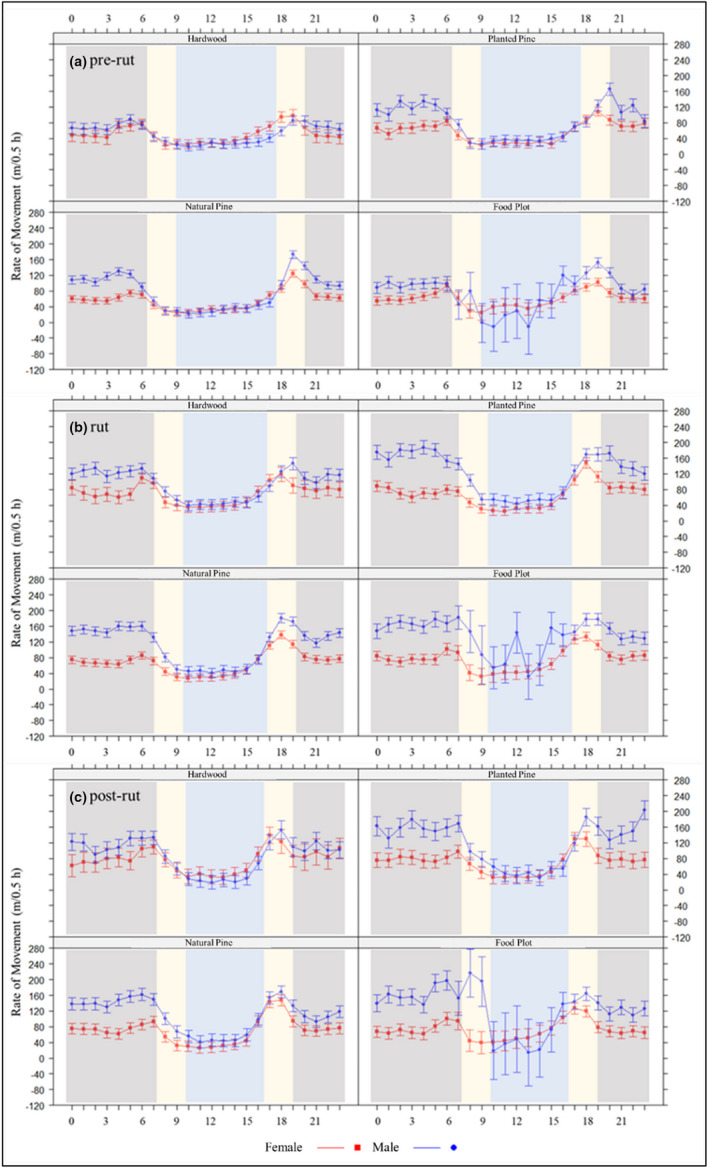
Effect of cover type, sex, hour of day, and season on white‐tailed deer (Odocoileus virginianus) movement rate from 2009 to 2018 in South Carolina, USA. Dark gray, beige, and light blue bands are considered night, dawn or dusk, and day, respectively. Error bars represent 95% confidence intervals.

Our top rate of movement model included a four‐way interaction among cover type, sex, time of day, and season (Table 3). On average, movement rate across cover types was greater for males (100 m/0.5 h) than for females (72 m/0.5 h; Figure 2; Table 4). Day movement rate was greatest in food plots compared to other cover types for both sexes (male = 85 m/0.5 h; female = 60 m/0.5 h). Day movement rate was least in hardwoods for males (33 m/0.5 h) and in planted pines for females (35 m/0.5 h). Both sexes had greater movement rates at dusk (male: 129 m/0.5 h; female: 114 m/0.5 h) compared to dawn (male: 95 m/0.5 h; female: 55 m/0.5 h) across cover types. Nocturnal movement rate for females was relatively constant across cover types (range = 63–87 m/0.5 h), while nocturnal movement rate for males was considerably greater and more variable (range = 75–164 m/0.5 h; Table 4).

**TABLE 3:** Number of parameters (K), Akaike's Information Criterion (AIC_c_), difference from lowest AIC_c_ (ΔAIC_c_), and model weights (w) for candidate models used to predict the effects of cover type, sex, time of day, and period of the breeding season on average movement rate (m/0.5 h) of white‐tailed deer (*Odocoileus virginianus*) from 2009 to 2018 in South Carolina, USA.

Candidate model	K	AIC_c_	ΔAIC_c_	W
Cover type * sex * time * breeding season	99	3227041.65	0.00	1.00
Cover type * sex * time	35	3230342.50	3300.85	0.00
Cover type * time * breeding season	51	3231810.20	4768.55	0.00
Cover type + sex + time + breeding season	13	3233168.93	6127.28	0.00
Cover type + time + breeding season	12	3233181.54	6139.88	0.00
Cover type * time	19	3234186.60	7144.95	0.00
Cover type + sex + time	11	3235054.86	8013.21	0.00
Cover type * sex * breeding season	27	3250397.33	23355.68	0.00
Cover type + sex + breeding season	10	3252053.88	25012.23	0.00
Cover type + sex	8	3254308.62	27266.97	0.00
Null	4	3257340.69	30299.04	0.00

**TABLE 4:** Mean estimates (β), standard errors (SE), lower confidence limits (LCL), and upper confidence limits (UCL) predicting the effects of sex, period of the breeding season, time of day, and cover type on average movement rate (m/ 0.5 h) of white‐tailed deer (*Odocoileus virginianus*) from 2009 to 2018 in South Carolina, USA.

Sex	Season	Time	Cover type	β	SE	LCL	UCL
Male	Pre‐rut	Dawn	Food plot	59.57	13.51	33.08	86.05
			Hardwood	42.97	5.74	31.71	54.22
			Natural pine	42.78	5.74	31.53	54.04
			Planted pine	63.21	6.65	50.19	76.24
		Day	Food plot	72.47	9.99	52.88	92.06
			Hardwood	24.94	5.19	14.78	35.10
			Natural pine	27.40	5.16	17.30	37.50
			Planted pine	36.47	5.41	25.87	47.07
		Dusk	Food plot	132.27	6.98	118.59	145.96
			Hardwood	61.71	5.94	50.07	73.34
			Natural pine	121.68	5.62	110.67	132.69
			Planted pine	91.30	6.49	78.57	104.02
		Night	Food plot	90.37	5.36	79.87	100.86
			Hardwood	75.36	5.35	64.87	85.84
			Natural pine	110.34	5.08	100.39	120.29
			Planted pine	112.61	5.62	101.59	123.63
	Rut	Dawn	Food plot	166.11	12.81	141.01	191.21
			Hardwood	78.87	5.56	67.97	89.77
			Natural pine	93.46	5.36	82.95	103.96
			Planted pine	109.97	6.08	98.05	121.89
		Day	Food plot	111.07	9.25	92.93	129.21
			Hardwood	41.46	5.15	31.37	51.56
			Natural pine	48.96	5.09	38.98	58.94
			Planted pine	53.59	5.34	43.11	64.06
		Dusk	Food plot	165.87	6.46	153.21	178.52
			Hardwood	106.36	5.87	94.85	117.87
			Natural pine	161.25	5.36	150.74	171.75
			Planted pine	151.80	6.08	139.88	163.72
		Night	Food plot	150.42	5.31	140.01	160.82
			Hardwood	116.06	5.22	105.82	126.29
			Natural pine	144.85	5.02	135.02	154.68
			Planted pine	163.64	5.36	153.15	174.14
	Post‐rut	Dawn	Food plot	198.56	18.46	162.38	234.75
			Hardwood	86.23	5.87	74.74	97.73
			Natural pine	93.67	5.49	82.92	104.43
			Planted pine	104.53	6.74	91.31	117.75
		Day	Food plot	72.03	12.82	46.90	97.16
			Hardwood	32.95	5.34	22.49	43.41
			Natural pine	48.46	5.17	38.33	58.60
			Planted pine	47.52	5.66	36.42	58.62
		Dusk	Food plot	155.09	6.33	142.68	167.50
			Hardwood	117.86	6.57	104.98	130.74
			Natural pine	150.71	5.50	139.93	161.50
			Planted pine	130.09	7.01	116.36	143.82
		Night	Food plot	140.93	5.32	130.50	151.36
			Hardwood	121.01	5.37	110.49	131.54
			Natural pine	131.08	5.04	121.20	140.97
			Planted pine	158.95	5.55	148.07	169.84
Female	Pre‐rut	Dawn	Food plot	53.46	7.67	38.43	68.50
			Hardwood	43.91	5.84	32.45	55.36
			Natural pine	42.26	4.92	32.63	51.89
			Planted pine	47.25	5.60	36.27	58.22
		Day	Food plot	50.47	5.31	40.07	60.88
			Hardwood	39.46	4.99	29.69	49.23
			Natural pine	34.64	4.72	25.39	43.90
			Planted pine	34.30	4.93	24.65	43.96
		Dusk	Food plot	96.47	5.33	86.01	106.92
			Hardwood	92.80	6.65	79.78	105.83
			Natural pine	100.64	4.99	90.85	110.43
			Planted pine	95.48	5.67	84.37	106.60
		Night	Food plot	62.60	4.86	53.08	72.11
			Hardwood	63.42	5.38	52.88	73.96
			Natural pine	67.61	4.69	58.42	76.80
			Planted pine	77.13	5.04	67.25	87.01
	Rut	Dawn	Food plot	65.32	7.22	51.16	79.48
			Hardwood	67.26	5.54	56.40	78.11
			Natural pine	55.05	4.83	45.58	64.51
			Planted pine	56.86	5.20	46.66	67.06
		Day	Food plot	62.58	5.24	52.31	72.84
			Hardwood	44.35	4.98	34.59	54.11
			Natural pine	40.62	4.70	31.41	49.82
			Planted pine	37.19	4.84	27.69	46.68
		Dusk	Food plot	130.11	5.24	119.85	140.37
			Hardwood	108.30	6.36	95.83	120.77
			Natural pine	127.23	4.85	117.73	136.72
			Planted pine	125.06	5.51	114.25	135.87
		Night	Food plot	82.18	4.79	72.79	91.57
			Hardwood	80.66	5.12	70.63	90.69
			Natural pine	76.36	4.66	67.23	85.50
			Planted pine	79.79	4.86	70.27	89.32
	Post‐rut	Dawn	Food plot	51.49	8.67	34.50	68.49
			Hardwood	65.82	6.20	53.67	77.98
			Natural pine	53.30	4.96	43.58	63.02
			Planted pine	59.19	5.49	48.42	69.96
		Day	Food plot	68.30	5.65	57.23	79.36
			Hardwood	38.44	5.28	28.10	48.79
			Natural pine	34.95	4.76	25.62	44.28
			Planted pine	34.14	4.92	24.49	43.79
		Dusk	Food plot	120.57	5.14	110.49	130.65
			Hardwood	119.14	7.18	105.06	133.22
			Natural pine	137.04	5.06	127.12	146.96
			Planted pine	116.08	5.81	104.69	127.47
		Night	Food plot	70.10	4.75	60.78	79.41
			Hardwood	86.62	5.48	75.89	97.35
			Natural pine	74.24	4.69	65.06	83.43
			Planted pine	77.39	4.96	67.68	87.10

**FIGURE 3:**
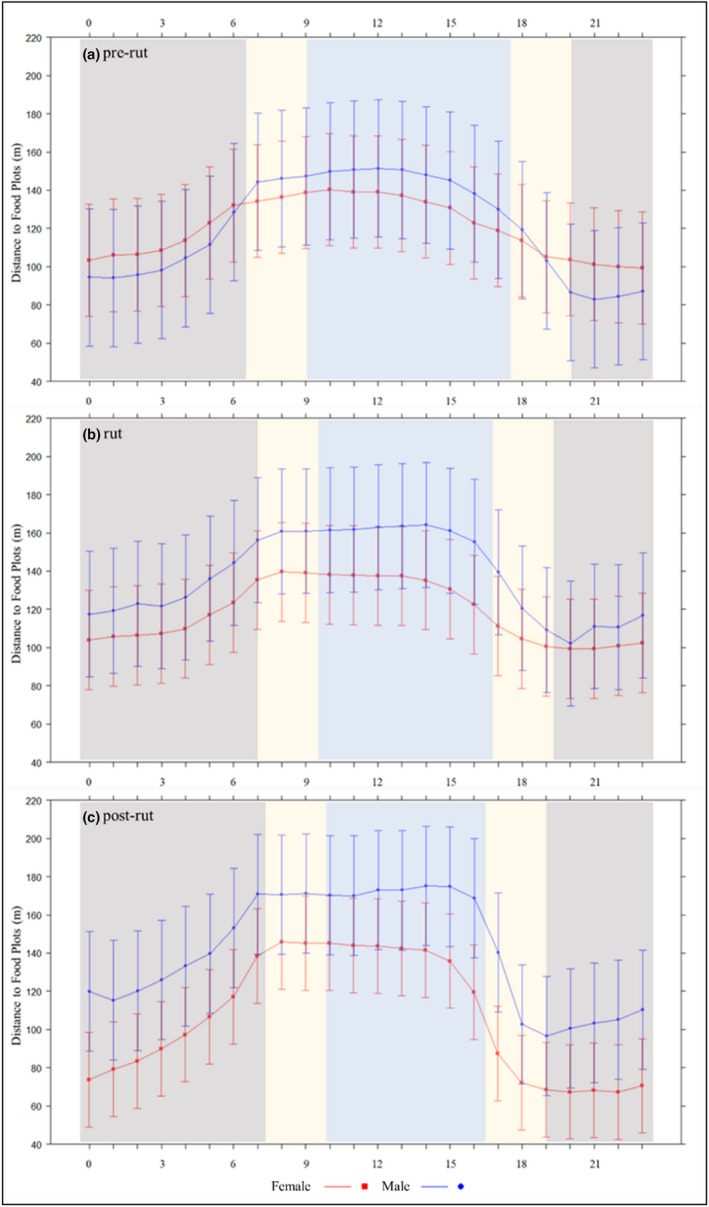
Effect of sex, hour of day (h = 0–23), and season on distance of white‐tailed deer (Odocoileus virginianus) to food plots from 2009 to 2018 in South Carolina, USA. Dark gray, beige, and light blue bands are considered night, dawn or dusk, and day, respectively. Error bars represent 95% confidence intervals.

**FIGURE 4:**
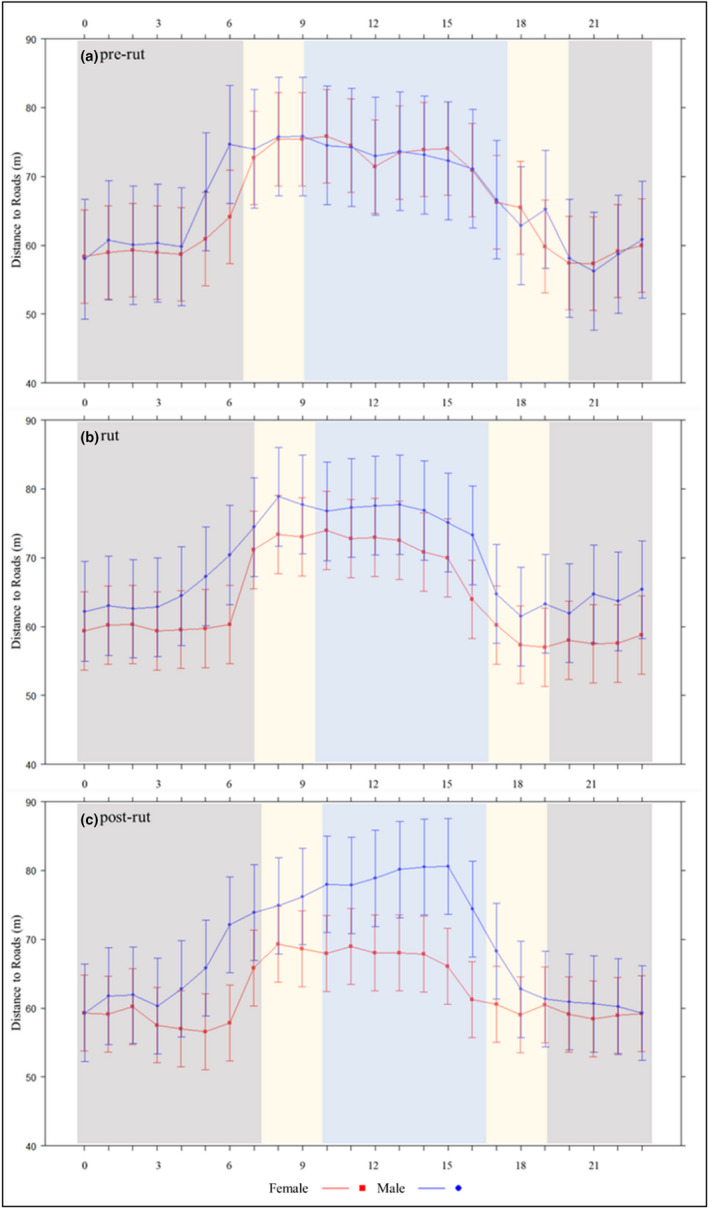
Effect of sex, hour of day (h = 0–23), and season on distance of white‐tailed deer (Odocoileus virginianus) to roads from 2009 to 2018 in South Carolina, USA. Dark gray, beige, and light blue bands are considered night, dawn or dusk, and day, respectively. Error bars represent 95% confidence intervals.

Our top models for minimum distance to food plots and roads (i.e., risky areas) included a three‐way interaction among sex, time of day, and season (Table 5). Both sexes tended to be closer to food plots and roads at night, but males showed greater avoidance of food plots and roads during the day (Figures 3 and 4). Both sexes were closer to food plots (male: 123 m; female: 101 m) and roads (male: 65 m; female: 60 m) at dusk compared to dawn (food plots: male 158 m, female 139 m; roads: male 76 m, female 71 m). Although statistically significant, likely due to sample size, the difference in distance to risky areas between sexes and periods was unlikely biologically significant (i.e., a few meters; Tables 6 and 7).

**TABLE 5:** Number of parameters (K), Akaike's Information Criterion (AIC_c_), difference from lowest AIC_c_ (ΔAIC_c_), and model weights (w) for candidate models used to predict the effects of sex, time of day, and period of the breeding season on the minimum distance of white‐tailed deer (*Odocoileus virginianus*) to food plots and roads from 2009 to 2018 in South Carolina, USA.

Candidate Model	K	AIC_c_	ΔAIC_c_	W
Food plots				
Sex * time * breeding season	27	3424466.19	0.00	1.00
Sex + time + breeding season	10	3427395.67	2929.48	0.00
Sex * time	11	3427699.08	3232.89	0.00
Sex + time	8	3427889.31	3423.13	0.00
Sex * breeding season	9	3439813.59	15347.41	0.00
Sex + breeding season	7	3441331.76	16865.58	0.00
Null	4	3441732.93	17266.74	0.00
Roads				
Sex * time * breeding season	27	3037914.55	0.00	1.00
Sex + time + breeding season	10	3038262.24	347.69	0.00
Sex * time	11	3038270.04	355.49	0.00
Sex + time	8	3038272.83	358.28	0.00
Sex * breeding season	9	3043256.60	5342.04	0.00
Sex + breeding season	7	3043433.99	5519.44	0.00
Null	4	3043491.25	5576.70	0.00

**TABLE 6:** Mean estimates (β), standard errors (SE), lower confidence limits (LCL), and upper confidence limits (UCL) predicting the effects of sex, period of the breeding season, and time of day on minimum distance to food plots and road (m) and 95% confidence intervals of white‐tailed deer (*Odocoileus virginianus*) from 2009 to 2018 in South Carolina, USA.

Sex	Season	Time	β	SE	LCL	UCL
Male	Pre‐rut	Dawn	142.99	16.17	111.29	174.68
		Day	145.63	16.12	114.04	177.21
		Dusk	112.38	16.16	80.69	144.06
		Night	92.65	16.11	61.07	124.23
	Rut	Dawn	159.13	16.14	127.50	190.77
		Day	161.21	16.11	129.64	192.79
		Dusk	126.25	16.14	94.62	157.89
		Night	120.13	16.11	88.57	151.70
	Post‐rut	Dawn	171.51	16.17	139.83	203.20
		Day	172.20	16.12	140.60	203.80
		Dusk	130.00	16.17	98.32	161.69
		Night	120.37	16.11	88.80	151.94
Female	Pre‐rut	Dawn	133.29	12.81	108.19	158.40
		Day	132.79	12.77	107.75	157.82
		Dusk	109.00	12.81	83.90	134.11
		Night	106.20	12.77	81.17	131.22
	Rut	Dawn	137.72	12.79	112.65	162.80
		Day	134.36	12.77	109.33	159.38
		Dusk	106.25	12.79	81.17	131.32
		Night	106.62	12.76	81.60	131.64
	Post‐rut	Dawn	144.70	12.81	119.59	169.82
		Day	140.79	12.78	115.75	165.84
		Dusk	86.27	12.81	61.16	111.38
		Night	84.61	12.77	59.58	109.63

**TABLE 7:** Mean estimates (β), standard errors (SE), lower confidence limits (LCL), and upper confidence limits (UCL) predicting the effects of sex, period of the breeding season, and time of day on minimum distance to roads (m) and 95% confidence intervals of white‐tailed deer (*Odocoileus virginianus*) from 2009 to 2018 in South Carolina, USA.

Sex	Season	Time	β	SE	LCL	UCL
Male	Pre‐rut	Dawn	75.72	3.48	68.90	82.53
		Day	73.01	3.41	66.32	79.69
		Dusk	64.20	3.47	57.40	71.00
		Night	60.55	3.40	53.88	67.23
	Rut	Dawn	76.79	3.44	70.04	83.54
		Day	76.35	3.40	69.67	83.02
		Dusk	62.99	3.44	56.24	69.74
		Night	64.42	3.40	57.76	71.08
	Post‐rut	Dawn	74.99	3.47	68.18	81.79
		Day	79.13	3.42	72.43	85.83
		Dusk	66.99	3.47	60.18	73.79
		Night	62.71	3.40	56.04	69.37
Female	Pre‐rut	Dawn	72.44	2.75	67.05	77.82
		Day	73.38	2.70	68.08	78.67
		Dusk	63.33	2.75	57.95	68.71
		Night	59.15	2.70	53.87	64.44
	Rut	Dawn	72.33	2.73	66.98	77.67
		Day	70.98	2.70	65.70	76.27
		Dusk	58.00	2.73	52.66	63.34
		Night	59.05	2.69	53.78	64.32
	Post‐rut	Dawn	69.01	2.75	63.61	74.40
		Day	67.37	2.71	62.06	72.68
		Dusk	59.81	2.75	54.42	65.19
		Night	58.74	2.69	53.46	64.03

## 4 DISCUSSION

Our results were consistent with previous evidence that white‐tailed deer shift space use to less intensively hunted areas (Byrne et al., 2014; Karns et al., 2012; Kilpatrick & Lima, 1999; Little et al., 2014; Little et al., 2016), or those providing greater concealment (Henderson et al., 2020; Naugle et al., 1997; Rhoads et al., 2013) during daylight hours. The hardwood drains on our study area were both areas of low hunting pressure and provided concealment cover. Specifically, they consisted of dense forests with abundant woody vegetation in the mid‐ and understory, and represented only 4.5% of the area in which a deer would be viewable from hunting stands. These areas also provided acorns and woody browse as forage during the study period. The planted pine cover type was similarly dense, had the second lowest visibility from hunting stands, and was also selected by deer during the day (slightly more so by females). Movement rates were low in these cover types during the day, especially hardwoods, indicating deer were likely bedded in them (Tables 4, 5, 6, 7).

Conversely, both sexes selected for food plots at night. Others have reported similar findings for males (Byrne et al., 2014; Karns et al., 2012), females (Larson et al., 1978), or both sexes (Montgomery, 1963). Deer use food plots because they often provide significantly greater forage density than the surrounding forest (Edwards et al., 2004; Lashley et al., 2011). Avoidance of food plots during the day may have been related to the hunting pressure they received. Specifically, food plots represented only 6% of the landscape, but 23% of the area in which a deer was viewable from a hunting stand. In contrast, another study reported that deer moved from intensively hunted forested areas to unhunted fields during the day in an area where hunters were only present in the forest (Sparrowe & Springer, 1970).

In contrast to the other cover types, there were no clear trends in deer selection or movement rates for the natural pine cover type, which consisted of frequently burned, open‐canopy longleaf and loblolly pine stands. Accordingly, the natural pine understory was dominated by low‐growing grasses and forbs. Forbs provide high quality forage for deer during spring and summer, but their availability and representation in the diet decreases during fall and winter, being replaced primarily by browse (Thill & Martin Jr, 1986). Natural pine was also present in the area surrounding hunting stands proportionate to its availability across the study area. Therefore, we believe forage availability and risk were less in natural pine compared to food plots, but predation risk in natural pine was still greater than in hardwoods or planted pines due to the limited concealment cover it provided compared to those cover types.

Perhaps our most interesting observation was that, although both sexes tended to use food plots more at night, female selection for food plots during all periods of legal hunting hours (i.e., dawn, day, and dusk) was greater than for males, and females also tended to be closer to risky areas (i.e., food plots and roads) during the day. This supports our original hypothesis that females would be more likely to use risky, forage‐rich areas during the day. Beier and McCullough (1990) similarly reported that female white‐tailed deer on the George Reserve in Michigan exhibited greater use of open cover types, but did not distinguish sex‐specific differences in cover type selection by diel period. Females require higher quality diets because of their smaller size and increased forage passage rate, which decreases the nutrients they absorb per unit of forage consumed. In contrast, males have a larger rumen and decreased passage rate, which increases the nutrients they absorb, even from low‐quality food items (Berini & Badgley, 2017). Greater quality diet in females has been documented in multiple ungulate species (e.g., Barboza & Bowyer, 2000; du Toit, 2005; Post et al., 2001; Ruckstuhl, 1998), including white‐tailed deer (Beier, 1987; Luna et al., 2013).

However, sex‐specific dietary requirements were not sufficient to explain our observations, as female selection for food plots was greater at night, when hunters were not present. This is consistent with Bowyer (2004), who suggested that both the gastrocentric and predation risk models are necessary to explain sexual segregation. Our results also support the risky time hypothesis (Creel et al., 2008), but offer the most direct support for the activity budget hypothesis (Ruckstuhl & Neuhaus, 2002) for sexual segregation. Specifically, Ruckstuhl and Neuhaus (2002) concluded that intersex differences in activity budgets drive sexual segregation, with differences in predation risk and forage selection being additive factors. Importantly, our results demonstrate that this pattern holds true even during the breeding season, which is when white‐tailed deer are less sexually segregated than at any other time of the year (DeYoung & Miller, 2011).

Crawford et al. (2019) demonstrated that white‐tailed deer within the Florida panther's range avoided risky places (i.e., trails) during risky times (i.e., night, when panthers are active). Coyotes were the primary nonhuman predators of deer on our site and are generally most active at night (McClennen et al., 2001). If resource selection on our site was driven by behavioral responses to coyote predation risk, we should have observed a distinctly different pattern, with deer selecting food plots during the day and dense cover at night, especially given that coyotes select for open cover types like food plots at night (Hickman et al., 2016). There were several likely reasons for the lack of apparent deer response to nonhuman predators on our site. First, the majority of coyote predation is on fawns during summer (Kilgo et al., 2012; McCoy et al., 2013) and predation of adult females is rare (Kilgo et al., 2016, but see Chitwood et al., 2014), but increased vigilance in response to increasing coyote abundance has been documented for adult females during fall in our study region (Gulsby et al., 2018). However, overall coyote abundance, and thereby predation risk, on our site was probably low due to intensive trapping efforts, which resulted in relatively low rates of fawn predation by coyotes compared to other areas in the Southeast. Further, recent evidence has also demonstrated that white‐tailed deer are more than twice as likely to flee in response to sounds from humans than other predators, indicating that the effect of human presence, let alone hunting activity, may be the most important driver of deer behavior (Crawford et al., 2022).

Although we believe overall deer behavior and sex‐specific differences in selection for risky and nonrisky areas during daylight hours were primarily shaped by sex‐specific resource requirements and predation risk, there are some important caveats to our interpretation. One is the lack of data from nonhunted periods, which would allow insight into whether the timing or magnitude of resource selection differed between hunted and nonhunted periods, by sex. Another uncertainty is whether the perceived risk of hunter harvest differed between sexes on our study area. During the study period, hunters on the site harvested females at twice the rate of males. However, it is unlikely that deer can perceive sex‐specific differences in harvest rates, and the presence of hunters or harvest of other deer is sufficient to elicit avoidance of areas frequented by hunters, across sex‐age classes. Finally, hunting pressure on our study area was very low, making it uncertain whether it was sufficient to drive the sex‐specific patterns we observed. However, Crawford et al. (2022) reported that human voices alone were sufficient to elicit flight responses in white‐tailed deer, and humans were active throughout our study area during the day, engaging not only in hunting, but also in non‐consumptive outdoor recreation and land management activities. Considering these uncertainties, we are not clear whether the patterns we observed were actually driven by sex‐specific differences in resource requirements (and thereby greater willingness of females to use risky places during risky times), or if these are simply innate sex‐specific patterns exhibited by white‐tailed deer. However, given the behavioral plasticity exhibited by white‐tailed deer in response to various risk landscapes and predator communities, we believe that is unlikely. Nonetheless, further experimentation with well‐known and controlled risk factors would aid in understanding of sex‐specific resource selection in response to predation risk. However, our study contributes valuable information to the literature by describing sex‐specific resource selection by diel period on a site where males and females had access to the same resources within the same landscape of risk.

## AUTHOR CONTRIBUTIONS

**Dylan Stewart:** Conceptualization (equal); formal analysis (lead); investigation (equal); methodology (lead); visualization (lead); writing – original draft (equal); writing – review and editing (equal). **Stephen S. Ditchkoff:** Conceptualization (equal); formal analysis (equal); funding acquisition (lead); investigation (equal); methodology (equal); project administration (equal); resources (lead); supervision (equal); visualization (equal); writing – original draft (equal); writing – review and editing (equal). **Bret A. Collier:** Formal analysis (equal); methodology (equal); validation (equal); visualization (equal); writing – original draft (equal); writing – review and editing (equal). **William D. Gulsby:** Conceptualization (lead); formal analysis (equal); investigation (equal); methodology (equal); project administration (equal); supervision (lead); validation (equal); visualization (equal); writing – original draft (equal); writing – review and editing (equal).

## CONFLICT OF INTEREST

The authors have no competing interests to declare.

## Data Availability

The data that support the findings of this study are available in the Dryad data repository: https://doi.org/10.5061/dryad.fttdz08wj.
